# Mid-term effect of balloon aortic valvuloplasty on mitral regurgitation in aortic stenosis

**DOI:** 10.1186/s12947-020-00193-3

**Published:** 2020-04-13

**Authors:** Ryota Masaki, Masamichi Iwasaki, Hidekazu Tanaka, Tomoyo Hamana, Susumu Odajima, Wataru Fujimoto, Koji Kuroda, Yutaka Hatani, Takumi Inoue, Hiroshi Okamoto, Masanori Okuda, Takatoshi Hayashi, Ken-ichi Hirata

**Affiliations:** 1 Department of Cardiology, Hyogo Prefectural Awaji Medical Center, Sumoto, Japan; 2 Division of Cardiovascular Medicine, Department of Internal Medicine, Kobe University Graduate School of Medicine, 7-5-2, Kusunoki-cho, Chuo-ku, Kobe, 650-0017 Japan

**Keywords:** Balloon aortic valvuloplasty, Mitral regurgitation, Aortic stenosis

## Abstract

**Background:**

Balloon aortic valvuloplasty (BAV) offers an alternative to conventional aortic valve replacement in elderly and frail patients with severe aortic stenosis (AS) for whom there are no other effective options. We aimed to investigate the mid-term effect of BAV on mitral regurgitation (MR) in patients with severe AS.

**Methods:**

Our analysis was based on the data from 83 patients with severe AS (mean age, 86 ± 5 years; female, 68) treated using BAV. Echocardiography was performed before the procedure and at 1 and 3 months after. MR was quantified by measuring the MR jet area, with more-than-moderate MR being clinically significant.

**Results:**

Forty patients were classified in this group (MR group). Significant reduction of MR was observed in the MR group at 1 month and 3 months after procedure, with no improvement in patients in the non-MR group. At 3 months, 15 of the 40 patients in the MR group still had significant MR, with the change at 1 month in the left ventricular end-systolic dimension (OR: 1.36; 95% CI: 1.05–1.76; *P* = 0.022) and MR jet area (OR: 1.95; 95% CI: 1.16–3.29; *P* = 0.012) being predictive of persisting significant MR at 3 months after BAV. The prevalence of New York Heart Association functional class III or IV decreased at 1 and 3 months after BAV in both groups.

**Conclusions:**

BAV provides a useful therapeutic strategy for elderly patients with severe AS who are not candidates for surgical or transcatheter aortic valve replacement, especially in those with significant MR.

## Background

The incidence of aortic stenosis (AS) is increasing as a result of aging of the general population, with poor survival anticipated without aortic valve replacement once symptoms develop [1, 2]. Balloon aortic valvuloplasty (BAV) is offered as an alternative to conventional surgical aortic valve replacement (SAVR) or transcatheter aortic valve replacement (TAVR) for elderly and frail patients with severe AS, for whom there are no other effective options [3]. The current American College of Cardiology/American Heart Association guidelines state that BAV may be considered as a bridge-to-SAVR or -TAVR in patients with severe symptomatic AS (Class IIb, Level of Evidence C) [4]. Despite the current TAVR era, BAV can still be considered as a worthwhile treatment for severe AS, providing an urgent salvage option for patients with severe AS who are in cardiogenic shock and those who are not candidates for SAVR or TAVR, as well as providing a bridge-to-SAVR or -TAVR in patients in whom the benefit of valve replacement is uncertain and those with a temporary contraindication to valve replacement. Mitral regurgitation (MR) is commonly observed in patients with severe AS [5]. The resolution of AS, by means of SAVR or TAVR, leads to an immediate drop in left ventricular (LV) systolic pressure, which reduces the pressure gradient across the mitral valve and, thus, improves MR severity. On the other hand, the risk for mortality increases in patients with severe AS in whom MR fails to improve after SAVR or TAVR [6–8]. However, changes in MR after BAV and identification of patients with severe AS for whom BAV could be of benefit to decrease MR remain to be clarified. Accordingly, the aim of our study was to investigate the mid-term effect of BAV on MR in patients with severe AS.

## Methods

### Study population

We retrospectively studied 140 consecutive patients with severe AS who underwent BAV at Awaji Medical Center, between April 2014 and February 2018. BAV was indicated as a bridge-to-SAVR or -TAVR, as a treatment option for patients in whom SAVR or TAVR was not suitable due to severe comorbidity, for risk reduction by avoiding cardiac surgery, and for diagnostic purposes, to determine the implications of AS on patient’s symptoms. Our study was approved by the ethics committee of Awaji Medical Center (No. 30–59), and the need for patient consent was waived due to the retrospective design of the study.

### Echocardiography examination

Echocardiography was performed using commercially available ultrasound systems, namely the Aplio XG and Aplio Artida (Canon Medical Systems, Tochigi, Japan) and EPIQ7 (Philips Medical Systems, Andover, MA) systems. Echocardiography was performed before BAV, and at 1 and 3 months after BAV, with standard measurements obtained in accordance with the current guidelines of the American Society of Echocardiography/European Association of Cardiovascular Imaging [9]. For the assessment of AS, the maximal aortic jet velocity was recorded using multiple echo windows, with the window providing the highest velocity signal selected. The maximal and mean pressure gradients across the aortic valve were calculated using a modified Bernoulli equation, with the aortic valve area estimated using the continuity equation and normalized to the body surface area (AVAi). AS was defined based on the recommendations of the American Heart Association and American College of Cardiology, with severe AS defined in relation to the AVAi (< 0.6 cm^2^/m^2^) [4]. MR was quantified in the apical long-axis view by measuring the MR jet area at mid-systole [4]. The severity of MR was graded by the MR jet area relative to the left atria area, as follows: none or trace; mild (MR jet area < 20%); moderate (MR jet area 20–40%); or severe (MR jet area ≥ 40%). A more-than-moderate MR grade was considered clinically significant. In addition, the etiology of MR was classified into the following four groups, based on Carpentier’s functional classification [10]: Type I, II, IIIa and IIIb.

### BAV procedures

All procedures were performed under local anesthesia at the puncture site, using either an antegrade or retrograde approach. The antegrade trans-septal approach, using the INOUE balloon (TORAY, Japan), was performed as previously reported [11]. Briefly, the balloon devices were delivered using a 14 Fr catheter via the femoral vein, with temporary pacing delivered using an 6 Fr catheter in the opposite femoral vein. A snare catheter, introduced via the radial artery, was secured to an extra-stiff, 0.032 in., guidewire, passing from the right femoral vein through the right atrium, left atrium, and left ventricle, and then across the aortic valve, providing sufficient support to deliver and control the balloon device. Systemic arterial pressure was monitored using a 5 Fr pig tail catheter place in the ascending aorta via the other radial artery. Though the INOUE balloon was our first choice for the antegrade approach, in case of difficulty crossing the INOUE balloon, the VACS II (Osypka AG, Germany) or TYSHAK (NumED CANADA INC., Canada) were selected as an alternate. In the conventional retrograde arterial approach, either a VACS II, TYSHAK, MAXI LD (Cardinal health Japan, Japan), or MUSTANG (Boston Scientific Limited, Ireland) balloon was used, based on the surgeon’s preference. The selected balloon was advanced from the femoral artery. The AcuNav (Siemens Medical Solutions, USA) was introduced using an 8 Fr catheter in the jugular vein and used to guide atrial septum puncture, to observe the aortic valve during balloon inflation, and to monitor for complications, such as cardiac tamponade and aortic regurgitation. We performed contrast-enhanced multidetector computed tomography to measure the size and area of aortic annulus.

### Statistical analysis

Continuous variables were expressed as mean values and standard deviation for normally distributed data, and as the median and interquartile range for non-normally distributed data. Categorical variables were expressed as frequencies and percentages. Continuous parameters between subgroups were evaluated using Student’s t test, with proportional differences evaluated using Fisher’s exact test. Univariate logistic regression analysis was initially used to identify parameters associated with persisting significant MR at 3 months after BAV. Significant parameters were entered in a multivariate analysis, using a stepwise selection, to identify independent predictors of significant MR after BAV. The entry criterion for an individual item into the multivariable logistic regression model was *p* < 0.05. For all steps, a *p*-value < 0.05 was regarded as statistically significant. All analyses were performed using a commercially available software (MedCalc Statistical Software version 18.10, Mariakerke, Belgium).

## Results

### Patients’ clinical and baseline characteristics

**Fig. 1:**
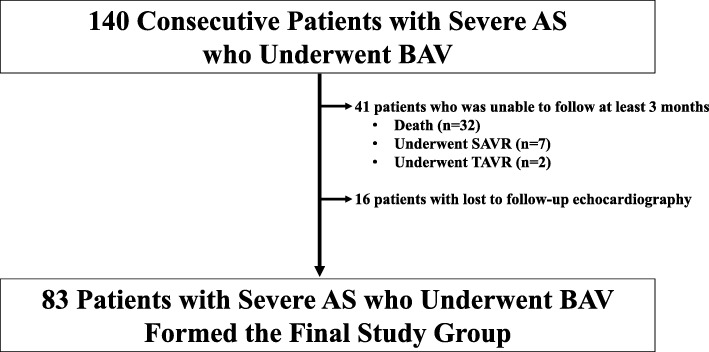
Clinical and baseline characteristics for patients with severe aortic stenosis (AS) treated using balloon aortic valvuloplasty (BAV) SAVR, aortic valve replacement; TAVR, transcatheter aortic valve replacement

BAV was successfully completed, without major complication, in all 140 patients included in the analysis. Of the initial group of 140 patients, 57 (40.7%) were not available for the 3 months follow-up due to death (*n* = 32), requiring subsequent SAVR (*n* = 7) or TAVR (*n* = 2), or lost to follow-up (*n* = 16). The echocardiography data at baseline and at 1 and 3 months after BAV, for the remaining 83 patients included in our final analysis are shown in Fig. 1. The indications for BAV included a bridge-to-SAVR or -TAVR (*n* = 27), the intervention of choice (n = 27), risk reduction associated with non-cardiac surgery (*n* = 17), and for diagnostic purposes (*n* = 12). The baseline clinical and echocardiographic characteristics of the 83 patients forming our study group are reported in Table 1, with key features summarized as follows: mean age, 86.2 ± 5.4 years; 56 women (67.5%); and mean LV ejection fraction (LVEF), 55.2 ± 11.2%. Severe aortic regurgitation was not identified in any of the patients before BAV.

**Table 1:** Baseline Clinical and Echocardiography Characteristics

Variables	All (*n* = 83)	MR group (*n* = 40)	Non-MR group (*n* = 43)	*P* value
**Clinical data**				
Age, years	86.2 ± 5.4	86.1 ± 6.5	86.4 ± 4.1	0.819
Female, *n* (%)	56 (67.5)	25 (62.5)	31 (72.1)	0.482
Body surface area, m^2^	1.4 ± 0.2	1.4 ± 0.2	1.4 ± 0.2	0.821
Body mass index, kg/m^2^	21.0 ± 3.4	20.8 ± 3.6	21.2 ± 3.3	0.623
NYHA III/IV, n (%)	47 (60.2)	28 (70.0)	19 (44.2)	0.094
STS score, %	9.6 ± 6.9	11.1 ± 7.5	8.2 ± 6.0	0.051
**Comorbidities,*n*(%)**				
Hypertension	63 (75.9)	29 (72.5)	34 (79.1)	0.609
Diabetes mellitus	24 (28.9)	13 (32.5)	11 (25.6)	0.629
Dyslipidemia	31 (37.3)	12 (30.0)	19 (44.2)	0.256
History of smoking	11 (13.3)	5 (12.5)	6 (14.0)	1.000
Coronary artery diseases	13 (15.7)	8 (20.0)	5 (11.6)	0.371
Peripheral arterial diseases	8 (9.6)	5 (12.5)	3 (7.0)	0.473
Cerebrovascular diseases	11 (13.3)	7 (17.5)	4 (9.3)	0.340
Atrial fibrillation	26 (31.3)	17 (42.5)	9 (20.9)	0.057
**Approach for BAV,*n*(%)**				
Antegrade BAV	65 (78.3)	29 (72.5)	36 (83.7)	0.288
Previous BAV	12 (14.5)	5 (12.5)	7 (16.3)	0.758
**Laboratory data**				
Hemoglobin, g/dL	11.0 ± 1.7	10.5 ± 1.7	11.6 ± 1.6	0.004
Albumin, g/dL	3.4 ± 0.5	3.2 ± 0.5	3.5 ± 0.5	0.010
Creatinine, mg/dL	1.0 (0.8–1.2)	1.1 (0.9–1.5)	0.9 (0.7–1.0)	0.002
eGFR, mL/min/1.73 m^2^	45.1 ± 20.3	39.4 ± 19.0	50.4 ± 20.1	0.012
BNP, pg/mL	377 (167–758)	702 (391–1042)	216 (112–422)	< 0.001
**Medications,*n*(%)**				
β-blocker	52 (62.7)	26 (65.0)	26 (60.5)	0.821
ACEI/ARB	57 (68.7)	26 (65.0)	31 (72.1)	0.636
Statin	33 (39.8)	12 (30.0)	21 (48.8)	0.116
CCB	34 (41.0)	9 (22.5)	25 (58.1)	0.002
Loop diuretics	48 (57.8)	25 (62.5)	23 (53.5)	0.506
MRA	22 (26.5)	11 (27.5)	11 (25.6)	1.000
Tolvaptan	6 (7.2)	5 (12.5)	1 (2.3)	0.101
Anticoagulant	15 (18.1)	8 (20.0)	7 (16.3)	0.778
Antiplatelet	22 (26.5)	11 (27.5)	11 (25.6)	1.000
**Echocardiography parameters**				
**Conventional parameters**				
LVEDD, mm	44.6 ± 6.7	46.2 ± 6.0	43.2 ± 7.1	0.039
LVESV, mm	30.7 ± 7.1	32.6 ± 6.8	28.9 ± 7.0	0.018
LVEF, %	55.2 ± 11.2	51.4 ± 13.0	58.6 ± 7.8	0.003
LVSVI, mL	41.6 ± 12.9	38.3 ± 11.7	44.7 ± 13.2	0.023
IVST, mm	10.8 ± 2.3	10.7 ± 2.1	10.9 ± 2.4	0.574
PWT, mm	10.5 ± 2.1	10.5 ± 2.0	10.5 ± 2.2	0.983
E/A	0.94 ± 0.58	1.1 ± 0.6	0.8 ± 0.6	0.089
E/e’	19.15 ± 8.21	20.37 ± 8.59	18.03 ± 7.78	0.203
TR-PG, mmHg	33.8 ± 14.2	40.5 ± 15.4	33.4 ± 12.2	0.023
AR ≥ moderate, *n* (%)	8 (9.6)	5 (12.5)	3 (7.0)	0.473
**AS parameters**				
AVA, cm^2^	0.68 ± 0.16	0.66 ± 0.17	0.70 ± 0.15	0.301
AVAi, cm^2^/m^2^	0.48 ± 0.11	0.47 ± 0.12	0.49 ± 0.10	0.401
Peak V, m/s	3.95 ± 0.85	3.81 ± 0.87	4.08 ± 0.81	0.147
mPG, mmHg	37.4 ± 16.3	35.6 ± 17.0	39.1 ± 15.6	0.330
**MR parameters**				
MR jet area, cm^2^	4.1 ± 3.6	7.0 ± 2.9	1.4 ± 1.2	< 0.001
%MR, %	20.6 ± 16.5	34.0 ± 13.1	8.1 ± 6.5	< 0.001
Etiology of MR				
Type I	16	16	–	–
Type II	5	5	–	–
Type IIIa	10	10	–	–
Type IIIb	9	9	–	–

Data are mean ± SD for normally distributed data and median and interquartile range for non-normally distributed data, or n (%)

*NYHA*, New York Heart Association, *BAV*, balloon aortic valvuloplasty, *eGFR*, estimated glomerular filtration rate, *BNP* brain natriuretic peptide, *ACEI* angiotensin converting enzyme inhibitor, *ARB* angiotensin II receptor blocker, *CCB* Calcium channel blocker, *MRA* mineral corticoid receptor antagonist, *LVEDD* left ventricular end-diastolic dimension, *LVESV* left ventricular end-systolic dimension, *LVEF* left ventricular ejection fraction, *LV* left ventricular, *SVI* stroke volume index, *IVST* interventricular septum thickness, *PWT* posterior wall thickness, *E* early diastolic wave velocity, *A* atrial wave velocity, *e’* early diastolic mitral annular velocity, *TR-PG* peak trans-tricuspid pressure gradient, *AS* aortic stenosis, *AVA* aortic valve area, *AVAi* indexed aortic valve area, *Peak V* peak trans-aortic velocity, *mPG* mean trans-aortic pressure gradient, *AR* aortic regurgitation, *MR* mitral regurgitation, *%MR* MR jet area of left atrial area

### Comparison of baseline characteristics between the MR and non-MR group

The MR group included 40 patients (48.2%) classified as having more-than-moderate MR, with the other 43 patients (51.2%) forming the non-MR group. The baseline clinical characteristics were similar for the MR and non-MR group, except that patients in the MR group were more likely to have a lower hemoglobin (10.5 ± 1.7 g/dL versus 11.6 ± 1.6 g/dL, *p* = 0.004), albumin (3.2 ± 0.5 g/dL versus 3.5 ± 0.5 g/dL, *p* = 0.010) and estimated glomerular filtration rate (39.4 ± 19.0 mL/min/1.73m^2^ versus 50.4 ± 20.1 mL/min/1.73m^2^, *p* = 0.012), and a higher creatinine (1.1 (1.0–1.2) mg/dL versus 0.9 (0.8–1.0) mg/dL, *p* = 0.002) and brain natriuretic peptide (BNP) (702 (574–876) pg/mL versus 216 (175–336) pg/mL, *p* < 0.001) levels. With regard to baseline echocardiography parameters, the two groups showed similar severity of AS, but with patients in the MR group being more likely to have a larger LV size (LV end-diastolic dimension, 46.2 ± 6.0 cm versus 43.2 ± 7.1 cm, *p* = 0.039; LV end-systolic dimension, 32.6 ± 6.8 cm versus 28.9 ± 7.0 cm, *p* = 0.018) and lower LVEF (51.4 ± 13.0% versus 58.6 ± 7.8%, *p* = 0.003).

### Time course of change in clinical, laboratory and echocardiography parameters after BAV

**Fig. 2:**
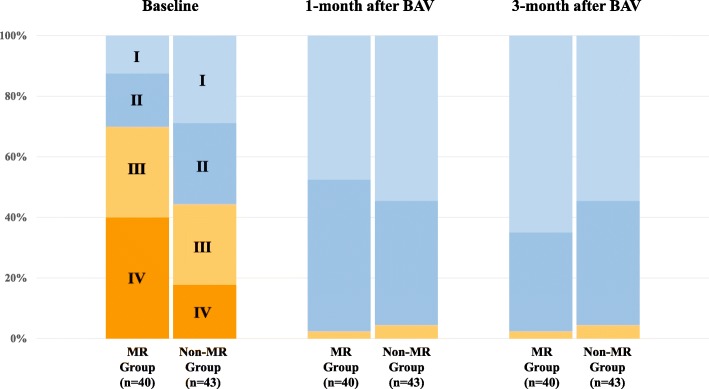
Change in the New York Heart Association (NYHA) functional class, from baseline, at 1 and 3 months after balloon aortic valvuloplasty (BAV) between patients in the significant mitral regurgitation (MR) and the non-MR group

The time course of change in clinical, laboratory, and echocardiography parameters after BAV is shown in Table 2 and Table 3. All parameters of AS severity significantly improved at 1 and 3 months after BAV, in both the MR and non-MR groups. The prevalence of the New York Heart Association (NYHA) functional class III or IV markedly decreased in both groups at 1 and 3 months after BAV (MR group: 70% at baseline versus 2.5% (1 month) and 2.5% (2 months); non-MR group: 44.2% versus 4.5% versus 4.5%, respectively; all *p* < 0.001). The changes in all NYHA functional class 1 month and 3 months after BAV in the MR and non-MR group are shown in Fig. 2. The BNP level significantly decreased in the MR group, both at 1 month (from 702 pg/mL to 421 pg/mL; *p* = 0.002) and 3 months after BAV (from 702 pg/mL to 281 pg/mL; *p* < 0.001), but not in the non-MR group. The LV size was significantly reduced at 3 months after BAV in the MR group (LV end-diastolic dimension, 46.2 ± 6.0 cm versus 44.0 ± 6.8 cm, *p* = 0.03; LV end-systolic dimension, 32.6 ± 6.8 cm versus 28.6 ± 7.0 cm, p < 0.001), but not in the non-MR group. The LVEF significantly improved at 3 months after BAV in both groups.

**Table 2:** Time Course of Parameters after BAV in the MR Group

	Baseline	1 month after BAV	P value	3 months after BAV	P value
**Clinical data**					
NYHA III/IV, n (%)	28 (70.0)	1 (2.5)	< 0.001	1 (2.5)	< 0.001
**Laboratory data**					
BNP, pg/mL	702 (391–1042)	421 (206–618)	0.002	281 (182–401)	< 0.001
**Conventional echo parameters**					
LVEDD, mm	46.2 ± 6.0	45.4 ± 6.5	0.237	44.0 ± 6.8	0.033
LVESD, mm	32.6 ± 6.8	31.2 ± 7.7	0.148	28.6 ± 7.0	< 0.001
LVEF, %	51.4 ± 13.0	54.1 ± 10.1	0.089	57.4 ± 12.5	0.007
LVSVI, mL	38.3 ± 11.7	41.3 ± 11.1	0.052	44.6 ± 11.2	0.002
E/A	1.0 (0.6–1.6)	0.7 (0.6–1.0)	0.003	0.7 (0.5–0.9)	0.001
E/e’	20.5 ± 8.7	15.5 ± 6.2	< 0.001	16.1 ± 7bnp.1	0.002
TR-PG, mmHg	40.5 ± 15.4	29.7 ± 8.4	< 0.001	28.9 ± 6.4	< 0.001
AR ≥ moderate, n (%)	5 (12.5)	6 (15.0)	1.000	4 (10.0)	1.000
**AS parameters**					
AVA, cm^2^	0.66 ± 0.17	0.84 ± 0.19	< 0.001	0.80 ± 0.20	0.001
AVAi, cm^2^/m^2^	0.47 ± 0.12	0.59 ± 0.12	< 0.001	0.57 ± 0.12	< 0.001
Peak V, m/s	61.0 ± 27.1	45.0 ± 19.1	< 0.001	51.7 ± 25.1	0.002
mPG (mmHg)	35.6 ± 17.0	24.9 ± 11.5	< 0.001	29.8 ± 15.2	0.003
**MR parameters**					
MR jet area, cm^2^	6.3 (4.5–9.7)	3.5 (1.5–7.4)	< 0.001	2.1 (1.1–6.0)	< 0.001
%MR, %	33.3 (22.6–42.7)	16.0 (8.9–34.3)	< 0.001	12.0 (4.7–29.4)	< 0.001
Mitral annular dimension, mm	28.5 ± 4.9	27.2 ± 4.2	0.030	25.5 ± 3.3	< 0.001
Tenting height, mm	4.1 ± 1.9	3.8 ± 1.9	0.307	2.8 ± 1.1	< 0.001
Tenting area, mm^2^	58.8 ± 31.6	52.0 ± 28.5	0.169	35.5 ± 14.5	< 0.001

**Table 3:** Time Course of Parameters after BAV in the Non-MR Group

	Baseline	1 month after BAV	P value	3 months after BAV	P value
**Clinical data**					
NYHA III/IV, n (%)	19 (44.2)	2 (4.5)	< 0.001	2 (4.5)	< 0.001
**Laboratory data**					
BNP, pg/mL	216 (112–422)	201 (103–405)	0.293	171 (11–368)	0.086
**Conventional echo parameters**					
LVEDD, mm	43.2 ± 7.1	42.8 ± 6.4	0.680	42.8 ± 6.4	0.659
LVESD, mm	28.9 ± 7.0	28.0 ± 6.9	0.230	27.4 ± 5.8	0.091
LVEF, %	58.6 ± 7.8	59.8 ± 8.8	0.318	61.4 ± 7.8	0.024
LVSVI, mL	44.7 ± 13.2	49.0 ± 13.5	0.016	49.3 ± 14.7	0.009
E/A	0.7 (0.5–0.9)	0.7 (0.5–0.9)	0.435	0.7 (0.5–0.8)	0.610
E/e’	17.8 ± 7.8	17.5 ± 8.2	0.692	18.0 ± 9.9	0.918
TR-PG, mmHg	33.4 ± 12.1	31.2 ± 13.5	0.138	30.6 ± 11.4	0.151
AR ≥ moderate, *n* (%)	3 (7.0)	4 (9.5)	0.713	6 (14.0)	0.483
**AS parameters**					
AVA, cm^2^	0.70 ± 0.15	0.88 ± 0.20	< 0.001	0.84 ± 0.20	< 0.001
AVAi, cm^2^/m^2^	0.48 ± 0.10	0.62 ± 0.14	< 0.001	0.59 ± 0.13	< 0.001
Peak V, m/s	69.1 ± 27.0	53.1 ± 23.1	< 0.001	57.0 ± 23.8	0.001
mPG (mmHg)	39.1 ± 15.6	30.9 ± 14.2	< 0.001	32.5 ± 14.3	< 0.001
**MR parameters**					
MR jet area, cm^2^	1.0 (0.3–2.5)	0.7 (0.2–1.8)	0.627	0.9 (0.3–2.1)	0.943
%MR, %	7.3 (2.0–12.9)	4.9 (1.2–11.3)	0.472	4.1 (1.7–12.2)	0.801
Mitral annular dimension, mm	25.8 ± 3.1	23.8 ± 3.4	0.001	24.7 ± 3.3	0.074
Tenting height, mm	3.6 ± 1.6	3.0 ± 1.5	0.047	3.0 ± 1.7	0.052
Tenting area, mm^2^	46.9 ± 24.0	36.5 ± 21.1	0.015	37.9 ± 25.9	0.052

Data are mean ± SD for normally distributed data and median and interquartile range for non-normally distributed data, or *n* (%)

Abbreviations as in Table 1

### Predictors of persisting significant MR at 3 months after BAV

**Fig. 3:**
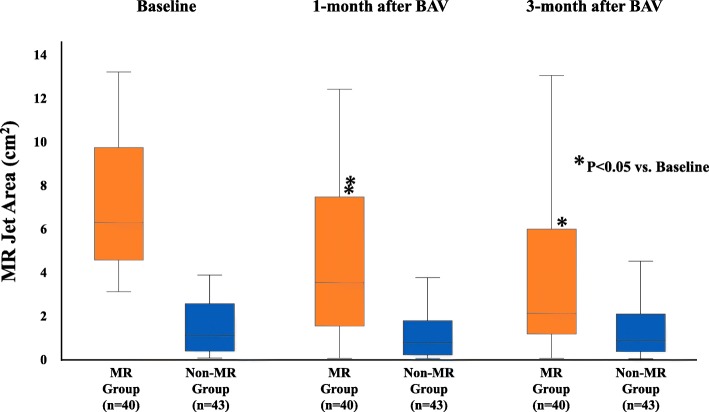
Change in mitral regurgitation (MR), from baseline, at 1 and 3 months after balloon aortic valvuloplasty (BAV) in the significant mitral regurgitation (MR) and the non-MR group, showing a significant improvement at both 1 and 3 months in the MR, but not the non-MR, group

**Fig. 4:**
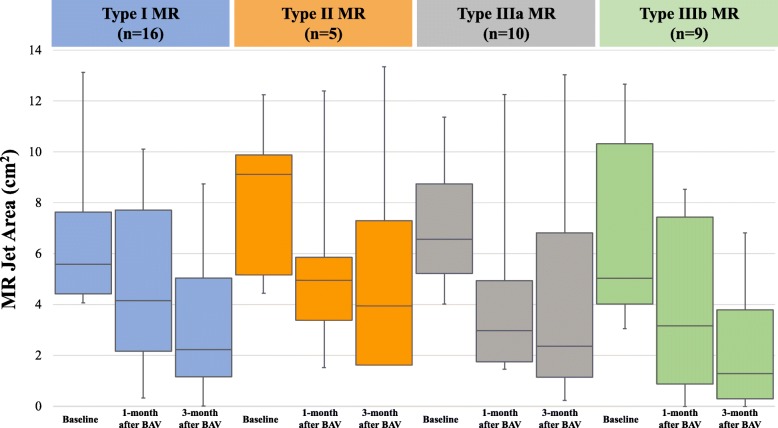
Change in mitral regurgitation (MR), from baseline, at 1 and 3 months after balloon aortic valvuloplasty (BAV) as a function of Carpentier’s functional classification in significant mitral regurgitation (MR) group, showing an overall tendency to improved MR after BAV, regardless of MR etiology, with the improvement being significant among patients with a Type I, II, and IIIb classification, after BAV

MR significantly improved both 1 and 3 months after BAV in the MR group, but not in the non-MR group (Fig. 3). In the MR group, the MR jet area at baseline was 6.3 cm^2^(4.5–9.7 cm^2^), and which gradually decreased at 1 month (3.5 cm^2^, 1.5–7.4 cm^2^) and at 3 months (2.1 cm^2^,1.1–6.0 cm^2^) after BAV. In addition, MR tended to improve after BAV regardless of the etiology of MR in the MR group, with the improvement being significant for patients in every etiology of MR (Fig. 4). At 3 months after BAV, significant MR persisted in 15 patients in the MR group. In 40 patients of the MR group, 25 patients (62.5%) improved MR jet area < 4.0 cm^2^ and 9 patients (22.5%) improved but remained significant MR, and 6 patients (15.0%) worsened MR compared with baseline. Categorized by MR etiology, persisted significant MR was showed 7/16 (43.8%) in Type I, 2/5 (40%) in Type II, 4/10 (40%) in Type IIIa, and 2/9 (22.2%) in Type IIIb. Though Type IIIb were tended to achieve much more reduction of MR, there was no significant difference among each etiology groups. The odds ratio (OR) and 95% confidence interval (CI) for univariate and multivariate logistic regression analysis of the variables associated with a persisting significant MR are summarized in Table 4. On multivariate logistic regression analysis, the change at 1 month, from baseline, in the LV end-systolic dimension (OR: 1.36; 95% CI: 1.05–1.76; *P* = 0.022) and MR jet area (OR: 1.95; 95% CI: 1.16–3.29; *P* = 0.012) were retained as independent predictive factors of persisting significant MR at 3 months after BAV.

**Table 4:** Univariate and Multivariate Analysis for Predicting Remaining Significant MR at 3-Month after BAV

Variables	Univariate analysis			Multivariate analysis		
	OR	95% CI	*P* value	OR	95% CI	*P* value
**Baseline parameters**						
Age	1.00	0.91–1.11	0.940			
Female	1.86	0.50–6.94	0.356			
BNP	1.00	1.00–1.00	0.130			
NYHA III/IV	1.29	0.31–5.35	0.722			
LVESD	0.96	0.87–1.06	0.469			
TR-PG	0.98	0.94–1.03	0.447			
AVAi	9.80	0.04–2225.02	0.410			
LVEF	1.03	0.98–1.09	0.253			
MR jet area	1.21	0.96–1.53	0.105			
E/e’	1.01	0.93–1.09	0.867			
**Change between baseline and 1-month after BAV**						
△LVESD	1.20	1.04–1.40	0.015	1.36	1.05–1.76	0.022
△TR-PG	1.05	1.00–1.11	0.067			
△MR jet area	1.31	1.05–1.62	0.016	1.95	1.16–3.29	0.012
△AVAi	0.04	0.00–130.06	0.443			
△LVEF	0.99	0.93–1.06	0.835			
△E/e’	1.03	0.94–1.12	0.510			

Abbreviations as in Table 1 and 2

## Discussion

We report significant improvement in MR after BAV in patients with severe AS, with the change in LV end-systolic dimension 1 month after BAV, from baseline, being independently associated with remaining significant MR 3 months after BAV. In addition, improvement in heart failure status after BAV tended to be more prominent among patients with severe AS and significant MR.

### Place of BAV in the TAVR era

BAV was first proposed in 1986, offering an alternative to conventional SAVR in elderly and frail patients with severe AS for whom there were no other effective options [3]. However, the hemodynamic improvement was reported to be short-lived, with rapid restenosis, with treatment outcomes being as poor as for patients not treated for severe AS [3, 12, 13]. Despite these limitations, BAV as a possible therapeutic intervention is considered for patients with severe AS and significant comorbidities which limits the use of a surgical cardiac intervention, such as chronic kidney disease, peripheral vascular disease, and coronary artery disease. With the aging of the general population, there is an increasing prevalence of elderly and frail patients presenting with severe AS for whom BAV can provide a beneficial therapeutic intervention, expanding the indications for BAV, despite the TAVR era [14]. In this study, we demonstrate that good clinical outcomes can be achieved at 1 and 3 months after BAV, including an improvement in cardiac status among patients with severe AS and significant MR.

### MR in patients with severe AS

MR is commonly observed in patients with severe AS [5]. In patients with severe AS, the mitral annulus, leaflets, and sub-valvular apparatus are often calcified, to varying degrees, while the LV size and function are typically normal. Occasionally, patients with AS are observed to have mitral valve prolapse or a flail leaflet, which are normally associated with mitral annular calcification. Ideally, simultaneous surgical replacement of both the aortic and mitral valve would be the preferred treatment for severe AS and significant MR; however, this surgical strategy increases the risk of morbidity and mortality, especially for elderly patients [15]. Whereas isolated SAVR in elderly patients is associated with an acceptable mortality rate, the surgical risk is significantly increased when double valve surgery is performed, with or without revascularization. The Euro Heart Survey on Valvular Heart Disease, in fact, reported a perioperative mortality rate of 6.5% for double valve intervention, compared to 2.7% for isolated SAVR and 4.3% for SAVR combined with revascularization [16].

The resolution of AS by means of SAVR or TAVR leads to an immediate drop in the LV systolic pressure, which reduces the pressure gradient across the mitral valve and, therefore, should improve MR severity. In the presence of secondary MR with mitral valve tethering, the resolution of the AS can reduce the mitral tenting area in the acute phase, which in turn leads to a decrease in MR severity. Coutinho et al. reported a 4.9-fold increase in the risk of mortality for patients in whom MR failed to improve after isolated SAVR [6]. Harling et al. also reported poorer early and late outcomes associated with moderate-to-severe MR left untreated at the time of SAVR [7]. In addition, the PARTNER trial reported an improvement in MR in the majority of patients after SAVR and TAVR (69.4 and 57.7%, respectively), but with a worsening of MR severity in 2.8 and 5.8%, respectively, of patients treated using SAVR and TAVR [8]. Although hemodynamic success of SAVR or TAVR would be expected to improve MR severity, other factors can potentially negatively affect MR severity, with the following having been identified after TAVR: [17, 18] presence of atrial fibrillation; left bundle branch block or right ventricular pacing; ischemic wall motion abnormalities; and self-expanding valve with deep implant. However, the mechanism underlying changes in MR after SAVR or TAVR and identification of patients for whom SAVR or TAVR may be effective to improve MR remain uncertain. Furthermore, studies to date have not generally reported on the mechanism or etiology of MR in patients who have undergone SAVR or TAVR.

### Difference among the etiology of MR

In this study, the reduction of LV cavity was the predictor of MR improvement. The effect for Type I and IIIb were recognizable, but Type II and IIIa which were leaflet issues were unclear. Though we could not find the statistically difference among the MR etiology in this small number study, it seems to be tendency of poor response in Type II and IIIa (Fig. 4). On the other hand, even in Type II and IIIa cases showed significant decreasing of the MR severity at 1 month and 3 months after BAV. This finding can bring great clinical worth for too sick elderly patients with AS and MR because BAV is less invasive, easy-to-use and low cost treatment.

### Clinical implications

In this study, we report a significant improvement in MR after BAV in patients with severe AS, regardless of MR etiology. Importantly, improvement in heart failure status, such as BNP level or NYHA functional class, tended to be more prominent among patients with severe AS and significant baseline MR. Therefore, our findings support BAV as a potentially beneficial therapy for elderly patients with severe AS, and those with significant MR more specifically, who are not candidates for SAVR or TAVR because of comorbidity. In this study, we demonstrate that good efficacy for severe AS with MR can be achieved at 1 and 3 months after BAV. Moreover, the mortality rate even within 3 months after BAV was as high as 22.8% in this study, but most of them were non-cardiac death (78%).

### Limitations

This is a retrospective study, including a small number of patients from a single-center. Therefore, prospective studies, involving larger numbers of patients from different centers, are required to verify our findings. Moreover, the Awaji Medical Center is not currently accredited for TAVR; thus, > 40 BAVs are performed annually at our center, which may have introduced some bias in the indications for BAV for patients with severe AS. Our patients who were cardiogenic shock, acute decompensated heart failure or required non-cardiac surgery were good indication for BAV, otherwise Although elective cases in this study such as cardiogenic shock, acute decompensated heart failure, or immediately required non-cardiac surgery (62.1%) may undergo primary TAVR in the hospital accredited for TAVR, such patients were good indication for BAV as well.

## Conclusions

Significant improvement of MR was observed among patients with severe AS who underwent BAV. Improvement of patients’ heart failure status after BAV tended to be more prominent among patients with severe AS and significant MR. BAV can be useful as a therapeutic strategy for elderly patients with severe AS, especially those with significant MR, who are not candidates for SAVR or TAVR because of comorbidity.

## Data Availability

Authors do not wish to share their data. All the data published in original paper belongs to Awaji Medical Center.

## Abbreviations

AVAi: Aortic valve area estimated using the continuity equation and normalized to the body surface area
AS: Aortic stenosis
BAV: Balloon aortic valvuloplasty
BNP: Brain natriuretic peptide
NYHA: New York Heart Association
LV: Left ventricular
LVEF: Left ventricular ejection fraction
MR: Mitral regurgitation
SAVR: Surgical aortic valve replacement
TAVR: Transcatheter aortic valve replacement
